# Modification of a Defect-Based Fatigue Assessment Model for Al-Si-Cu Cast Alloys

**DOI:** 10.3390/ma11122546

**Published:** 2018-12-14

**Authors:** Roman Aigner, Martin Leitner, Michael Stoschka, Christian Hannesschläger, Thomas Wabro, Robert Ehart

**Affiliations:** 1 Christian Doppler Laboratory for Manufacturing Process based Component Design, Chair of Mechanical Engineering, Montanuniversität Leoben, 8700 Leoben, Austria; martin.leitner@unileoben.ac.at (M.L.); michael.stoschka@unileoben.ac.at (M.S.); 2 University of Applied Sciences Upper Austria, 4600 Wels, Austria; christian.hannesschlaeger@fh-wels.at; 3 BMW Motoren GmbH, 4400 Steyr, Austria; thomas.wabro@bmw.com (T.W.); robert.ehart@bmw.com (R.E.)

**Keywords:** aluminium casting, fatigue assessment, defects, statistical distribution, extreme value statistics, computed tomography

## Abstract

Cast parts usually inherit internal defects such as micro shrinkage pores due to the manufacturing process. In order to assess the fatigue behaviour in both finite-life and long-life fatigue regions, this paper scientifically contributes towards a defect-based fatigue design model. Extensive fatigue and fracture mechanical tests were conducted whereby the crack initiating defect size population was fractographically evaluated. Complementary in situ X-ray computed tomography scans before and during fatigue testing enabled an experimental estimation of the lifetime until crack initiation, acting as a significant input for the fatigue model. A commonly applied fatigue assessment approach introduced by Tiryakioglu was modified by incorporating the long crack threshold value, which additionally enabled the assessment of the fatigue strength in the long-life fatigue regime. The presented design concept was validated utilising the fatigue test results, which revealed a sound agreement between the experiments and the model. Only a minor deviation of up to about five percent in case of long-life fatigue strength and up to about 9% in case of finite-lifetime were determined. Thus, the provided extension of Tiryakioglu’s approach supports a unified fatigue strength assessment of cast aluminium alloys in both the finite- and long-life regimes.

## 1. Introduction

Aluminium cast alloys generally feature a proper relation of fatigue strength to density in terms of lightweight design. Furthermore, their excellent castability enables the manufacturing of rather complex geometries [1,2]. Hence, aluminium cast components are quite commonly used for demanding production parts, as in the case of automotive engines or electric drivetrain components. However, the manufacturing process itself implicates heterogeneous material properties due to varying local feeding and cooling rates. Additionally, the local cooling rate significantly influences the microstructural properties, such as the secondary dendrite arm spacing (DAS) [3]. Preliminary studies reveal that the local microstructure correlates well with the fatigue life [4,5,6]. The major influence is given by the statistical distribution of defects and their spatial extent, as exemplified in [7,8,9]. It has been shown that the local microstructure represented by the dendrite arm spacing value correlates well with the statistical size and distribution of inhomogeneities (e.g., micropores) [10]. In order to enhance the survival probabilities of crucial cast components, it is therefore inevitable to consider local pore size distributions in the fatigue design process. Preliminary studies revealed that the statistical distribution of fatigue-initiating defect sizes in cast parts can be described well by extreme value statistics such as the generalized extreme value (GEV) distribution and extreme value distribution of type one [11,12,13,14]. The latter is also referred to as Gumbel distribution and facilitates the assessment of maximum extreme values of the distribution—see Equation (1) [15]. If minimal values have to be taken into account, the inverse formulation can be applied:

(1) $$f\left(x\right)=\frac{1}{\delta }\exp\left(-\frac{x-\mu }{\delta }\right)\exp\left[-\exp\left(-\frac{x-\mu }{\delta }\right)\right],$$

with f(x) being the probability density function, its course is defined by the distribution parameters μ and δ, also called location and scale parameters. Tiryakioglu proposed a methodology to link the finite fatigue life with the cumulative distribution function of crack-initiating defect sizes [16,17]. By invoking the Paris–Erdoǵan law for stable crack growth, a proper relationship between the probability of failure $P_{f}$ and micropore size distribution can be established—see Equation (2) [18]:

(2) $$P_{f}=1-\exp\left\{-\exp\left[\frac{\mu }{\delta }-\frac{2}{\delta \sqrt{\pi }}{\left(\frac{N_{f}-N_{i}}{B\cdot \sigma_{a}^{-m}}\right)}^{\frac{2}{2-m}}\right]\right\}.$$

Hereby, μ and δ are the location and scale parameters of the Gumbel distribution of fracture initiating micropore sizes, represented by the equivalent circle diameter of the very defects $d_{eq}$, which can be evaluated based on the measured crack-initiating defect size area utilising Equation (3). Furthermore, Tiryakioglu’s model takes into account the load cycles until failure $N_{f}$ and until crack initiation $N_{i}$, the load stress amplitude $\sigma_{a}$, the crack propagation slope m and the offset parameter B. Nevertheless, due to the fact that this approach is based on the Paris–Erdoǵan law for stable crack growth, it is not valid for fatigue strength assessment in the long-life regime:

(3) $$d_{eq}=\frac{2}{\sqrt{\pi }}\cdot \sqrt{area}.$$

This methodology turned out to be well suited for assessing the finite-life of defect based fatigue failures [19]. Further information on this approach is given in [16]. Hence, the micropore sizes have to be subsequently investigated after fatigue testing by analysing the fracture surfaces, either by means of digital or scanning electron microscopy (SEM). One non-destructive methodology is the investigation of intrinsic flaws by X-ray computed tomography (XCT) scans [20]. This technology supports the three-dimensional assessment of inhomogeneities, in terms of their often complex shapes, orientations, and locations. Preliminary studies propose the number of load cycles until crack initiation $N_{i}$ is determined by the amount of cycles until fracture. The correlation factor is judged to be between 0.3 and 0.4 by [21]. Another micro-cell model to estimate $N_{i}$ is introduced in [22]. The authors of [23] propose the initiation lifetime to be significantly dependent on the microstructure, represented by the DAS and also of the very local stress condition near the flaw. With α and β being constants of the crack initiation model, the nominal stress amplitude $\sigma_{n,a}$ is amplified by a local stress concentration factor $k_{\sigma }$ (see Equation (4)).

(4) $$N_{i}=\frac{C_{0}}{DAS}{\left[\frac{1}{k_{\sigma }\sigma_{n,a}}\left(k_{0}+\frac{\alpha }{\sqrt{DAS}}\right)\right]}^{\frac{2}{\beta }}.$$

**Figure 1:**
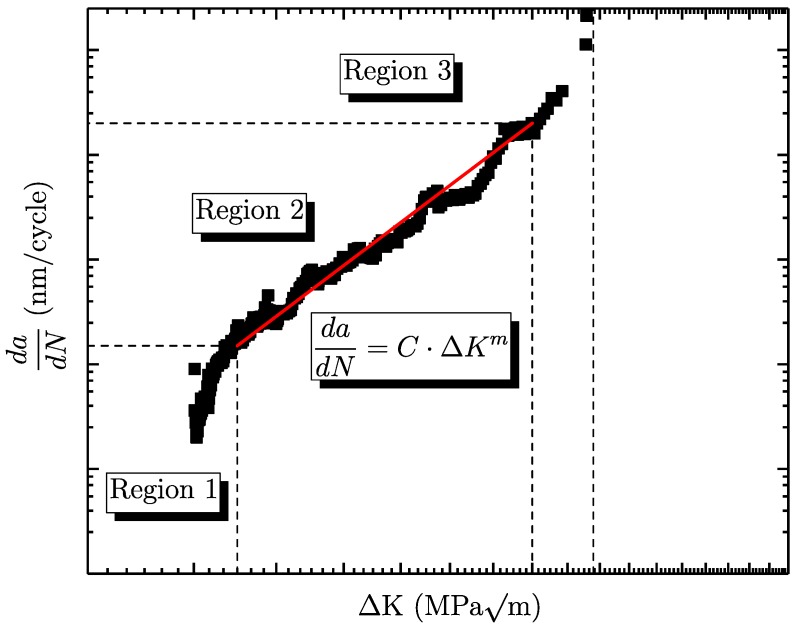
Representative crack–propagation curve with labelled regimes.

Different approaches can be used to characterise the fatigue crack growth. By plotting the crack growth per load cycle against the cyclic stress intensity factor, the typical S-shaped da/dN curve is obtained, as can be seen in Figure 1. The graph can be divided into three major regions. Region 1 is located on the left-hand side, characterising the short cracks and the transition to the long-crack area, where crack closure is built up. Preliminary studies investigated the impact of different microstructures on short and long crack growth (see [24]). It was found that the microstructure significantly impacts the long crack propagation, whereas the corresponding short crack values showed no major dependency. On the right-hand side of Figure 1, the zone of unstable crack growth, region 3, exists where the stress intensity increases towards the critical value $K_{I,c}$, as burst failure. Between these two boundaries, there is the area of stable crack growth, labelled as region 2. The most common model to characterise stable fatigue crack growth was presented by Paris–Erdoǵan [18] (see Equation (5)):

(5) $$\frac{da}{dN}=C\cdot \Delta K^{m},$$

with *C* being a material-dependent coefficient, ΔK the stress intensity range and *m* the slope of crack propagation. Klesnil and Lukas extended this approach including the long crack threshold $\Delta K_{th,lc}$ [25]. This model is capable of assessing the crack propagation curve in the regions 1 and 2 (see Equation (6)):

(6) $$\frac{da}{dN}=C\cdot \left(\Delta K^{m}-\Delta K_{th,lc}^{m}\right).$$

An elaborated approach to characterise all three regions of the crack propagation curve is given by Forman/Mettu, usually denoted as the NASGRO approach [26,27] (see Equation (7)). The crack opening function is in formula *f*, *n* is the slope of the crack propagation, *R* the load stress ratio, and *q* and *p* constants describe the rounded transition between the crack growth regions:

(7) $$\frac{da}{dN}=C{\left[\left(\frac{1-f}{1-R}\right)\Delta K\right]}^{n}\frac{{\left(1-\frac{\Delta K_{th,lc}}{\Delta K}\right)}^{p}}{{\left(1-\frac{\Delta K_{max}}{K_{c}}\right)}^{q}}.$$

To assess the fatigue strength in the long-life region $\sigma_{LLF}$, either Murakami’s $\sqrt{area}$ concept or the Kitagawa–Takahashi diagram with its modifications of El-Haddad and Chapetti are commonly applied [28,29,30,31,32]. Murakami’s approach is based on the coefficient $C_{1}$ depending on the defect location and the constant $C_{2}$, depending on the investigated material (see Equation (8)). The coefficient $C_{1}$ is proposed to be 1.56 for interior subsurface defects, and 1.43 for surface defects [33]. Hence, surface intersecting defects are considered to be more fatigue-sensitive, even in the presence of larger interior defects, which is in line with preliminary studies [34,35]. Additionally, the Vickers hardness (HV) of the assessed material is used as a base material strength parameter [36,37].

(8) $$\sigma_{LLF}=C_{1}\frac{HV+C_{2}}{{\sqrt{area}}^{1/6}}.$$

Applying this model, the alternating long-life fatigue resistance $\sigma_{LLF}$ is estimated at a total number of ten million cycles and is greatly affected by the effective micropore area. The defect size is evaluated as the projected flaw area perpendicular to the direction of the maximum principal normal stress [29]. On the one hand, preliminary studies [38] reveal a proper conformance with Murakami’s empirical approach, but the present model does not invoke the defect size distribution itself. As presented in preliminary studies [39,40,41,42], the statistical distribution of flaw sizes can be evaluated by non-destructive investigation of the defect population, such as X-ray computed tomography scanning. However, the statistical distribution of the most extreme values evaluated from the XCT does not always represent the distribution of fatigue fracture initiating defects, due to the fact that cracks may initiate at the surface near heterogeneities even in the presence of larger flaws within the bulk volume (see [43,44,45]). Thus, the flaw size as a sole valuation parameter is not applicable. By weighting heterogeneities with additional geometry factors, depending on their location, size and orientation [46], a much more reliable distribution of fatigue critical defect sizes can be assessed. Another study [47] reveals the relationship between the porosity, the microstructure and the ductile fracture behaviour, invoking image-based finite element analysis. Therefore, the authors in [47] observed the microstructure in an aluminium alloy by means of XCT, revealing a hydrogen-pore mechanism induced fracture, as strain localizes at extrinsic and intrinsic inhomogeneities. Tenkamp et al. [48] proposed a methodology to correlate defect sizes with the fatigue strength, invoking Kitagawa–Takahashi diagrams [49]. Hereby, the authors in [48] investigated the critical defect sizes by means of XCT scans. Afterwards, the initial crack length was estimated as the average of the 10% fraction of the largest evaluated equivalent pore diameter, which was revealed to meet the fractography results well. However, the application of the distribution of flaw sizes for fatigue assessment has still not yet reached its full potential. Existing defect based models just correlate the fatigue strength with the fatigue fracture initiating size of the very tested specimen. As the presented model is based on the distribution of the holistic sample location itself, the corresponding fatigue strength can be assessed statistically. Moreover, the approach in this work enables a statistical fatigue strength assessment which can be realised even early on in the design process by estimation of the defect distribution either by means of XCT scans or deduced by simulation of local cooling conditions. Hence, this paper scientifically contributes with the following research tasks:

- Experimental investigation of the impact of local defect population on the fatigue behaviour of Al-Si-Cu cast alloys.
- Estimation of crack initiation lifetime based on experimental analysis using in situ XCT scans.
- Extension of the fatigue lifetime model by Tiryakioglu [12] to additionally assess the fatigue strength in the long-life region.

## 2. Materials and Methods

The investigated material is a commonly used aluminium alloy EN AC-46200 with T6 heat treatment. The specimens are extracted from one batch of a gravity casted characteristic automotive part at two different locations, referred to as position A and position B. The nominal chemical composition of the investigated material is given in Table 1. Further information on the sampling positions is provided in a preceding study (see [19]). The T6 heat-treatment is separated into three stages and is known to significantly enhance mechanical properties, in terms of ductility and strength [50]. At first, the components are solution treated at high temperatures, in order to force the dissolution of Cu-rich intermetallic phases, since ${\mathrm{Al}}_{2}$Cu intermetallics dissolve at an exposure time of 30 min at an operating temperature of 510 ${}^{\circ }$C [51]. Thus, a homogeneous microstructure can be obtained after solidification. Secondly, the parts are quenched, usually at room temperature. Finally, the components are age hardened for maximal strength. This heat-treatment is also referred to as complete ageing [52]. The impact of T6 heat-treatment on the dendrite arm spacing, quasi-static properties and fatigue strength is given in [53,54].

In addition, the sample microstructures are investigated by means of extensive metallographical analysis. Due to the significantly varying local cooling rates, the sampling positions greatly differ in terms of microstructural properties. Hence, the different specimen conditions inherit significantly different defect populations, as the spatial extent of micropores correlates well with the local dendrite arm spacing (DAS). The DAS in the testing region of position A is estimated to be 26.40 ± 1.51 μm, where the DAS in position B is significantly greater with 53.26 ± 3.17 μm.

**Figure 2:**
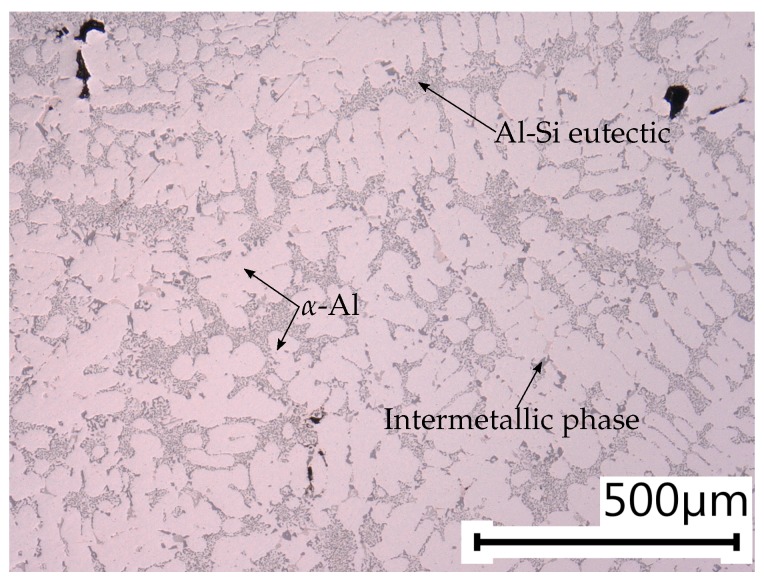
Representative metallographical specimen of position A with α-Al, Al-Si eutectic and intermetallic phases.

**Figure 3:**
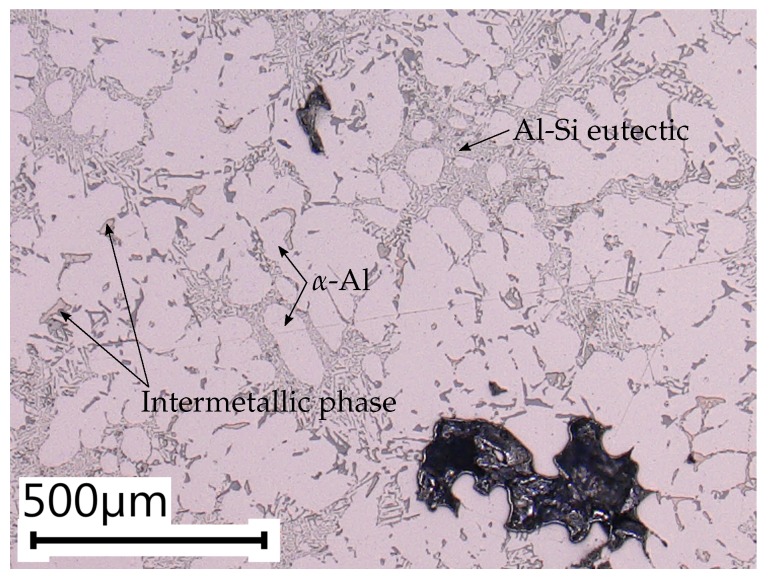
Representative metallographical specimen of position B with α-Al, Al-Si eutectic and intermetallic phases.

In Figure 2 and Figure 3, the local microstructures at the specimen locations A and B are displayed. As stated before, the local DAS in position B is more than two times the DAS value of specimen position A. Hence, the probability of larger defects is significantly enhanced in position B, which goes in line with results of previous studies [38,55] as well as with the subsequent fractographic analysis.

**Figure 4:**
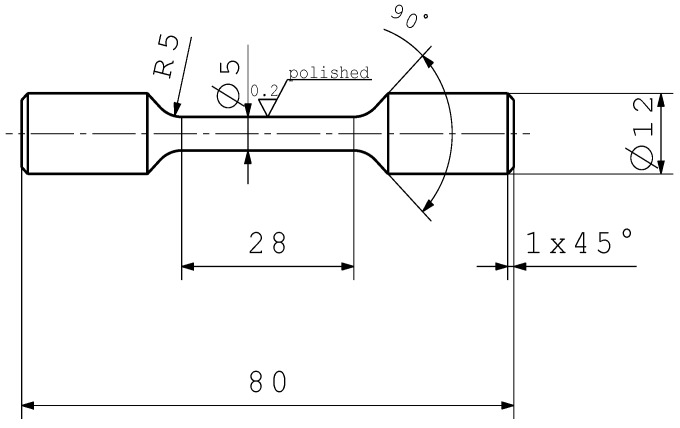
Tensile test specimen geometry.

The tensile test specimen geometry is depicted in Figure 4, respectively. In order to statistically evaluate the fatigue strength and the quasi-static properties, each test series contains a minimum of 15 HCF, 3 SENB, 3 tensile and 3 metallographic specimen. To reduce any roughness based effects such as micro-notches [56], every single specimen is polished prior to the testing. The tensile specimens are tested at a hydraulic Instron Schenk hydraulic strain-controlled system (Darmstadt, Germany) with a strain rate of $3.6\times 10^{-3}$ 1/s, utilising an extensometer. The fatigue tests are conducted at different specimen locations, utilising a Rumul electro-magnetic resonance testing machine (Neuhausen am Rheinfall, Switzerland). The testing frequency is about 108 Hz. Specimens are tested until burst failure, respectively a total runout number of load cycles of 1E7. The tests are executed at room temperature with an alternating tension/compression load at a load stress ratio of *R* = −1. The crack propagation tests are conducted at a Rumul electro-magnetic resonance bending test machine and incremental load growth at a load stress ratio of *R* = −1, in line with fatigue tests. The X-ray computed tomography investigations are carried out by a Phoenix/X-ray Nanotom 180 (Gelsenkirchen, Germany) and a voxel-size of just 5.5 μm.

## 3. Results

### 3.1. Fatigue and Quasi-Static Testing

As the investigated material exhibits a defect based failure criterion, it generally possesses no pronounced endurance limit. In a common guideline [57], a bilinear model for characterisation of an S–N curve is therefore proposed. It is suggested that the slope in the long-life region $k_{2}$ can be set to a scalar value of 25 in [57]. Nevertheless, preliminary studies revealed, that a correlation between the slopes in the finite-life $k_{1}$ and long-life region $k_{2}$ matches the fatigue test data of aluminium cast best. Therefore, a correlation of $k_{1}=5\times k_{2}$ is utilised for characterising the S–N test data, as proposed in [58]. In addition, the fatigue in the finite-life region is statistically evaluated, by means of [59]. The statistical investigation of fatigue scatter in the long-life region is performed by means of a statistical procedure given in [60]. All fatigue data is normalised by the fatigue strength of sample position A at ten million load cycles.

**Figure 5:**
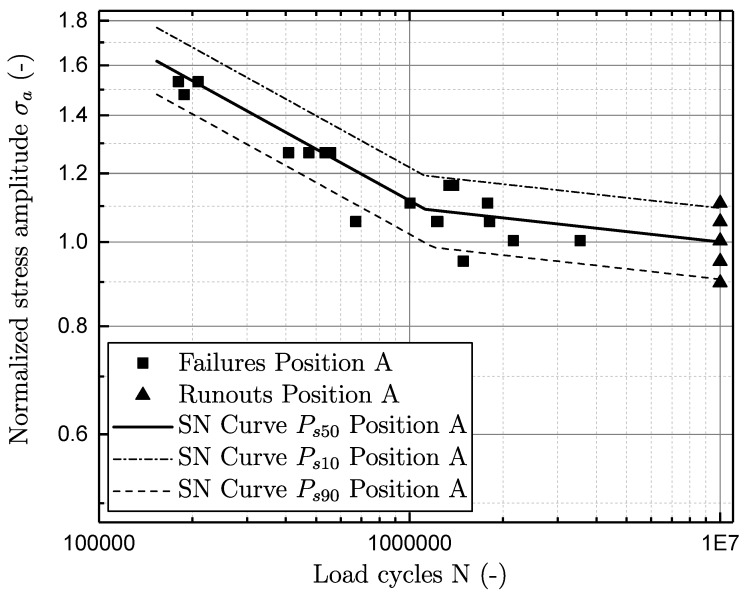
S–N curve for position A.

**Figure 6:**
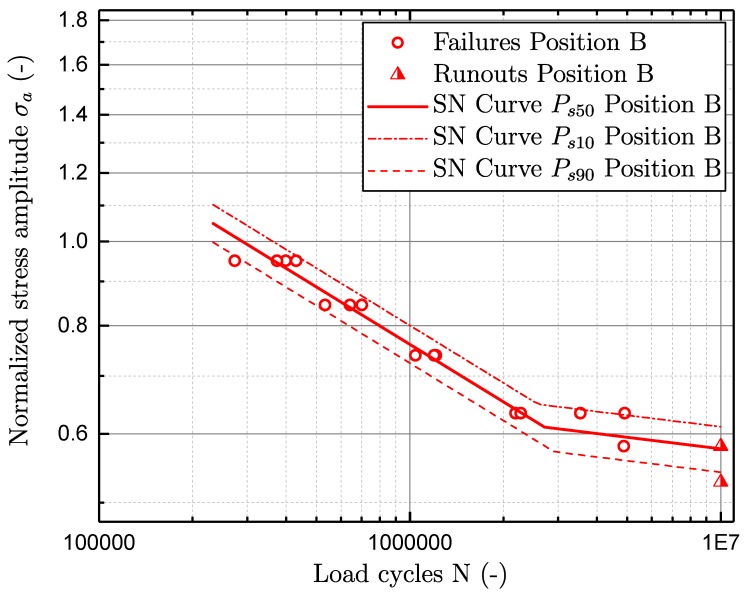
S–N curve for position B.

The effect of the local microstrucure is in line with the corresponding defect population and its effect on fatigue life is displayed in Figure 5 and Figure 6. Hence, the fatigue strength of specimen location B is significantly lower than for location A. The estimated data are listed in Table 2 and reveal the fatigue strength for position A to be nearly double that of the fatigue strength of position B. The statistical investigation of the specimen failures in the finite-lifetime proposes a slope $k_{1}$ of approximately 5 for both sampling positions. The second slope $k_{2}$ is therefore just slightly corrected regarding the proposed factor of 25 in [57]. In addition, the number of cycles $N_{k}$, locating the transition region of finite-lifetime to long-life region, is significantly increased in position B compared to position A.

**Figure 7:**
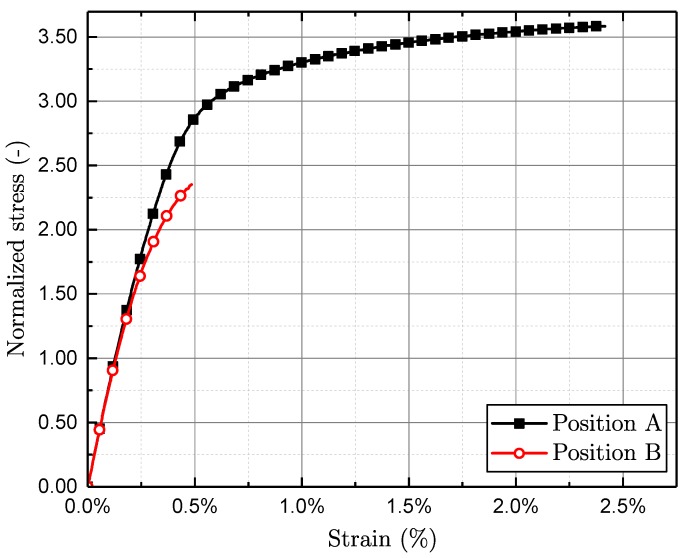
Representative tensile tests for positions A and B.

In addition, the quasi-static properties are investigated by means of tensile tests. Representative tensile test results for positions A and B are displayed in Figure 7.

All tests are conducted at room temperature utilising a strain controlled experimental procedure at a strain rate of $3.6\times 10^{-3}$ 1/s. All quasi static data are subsequently investigated by means of a common guideline [61]. The listed yield strength in Table 3 is evaluated at a strain value of 0.01% due to the quite brittle material behaviour in position B. The quasi-static test results of the tensile tests agree with the fatigue data, as lower dendrite arm spacing correlates well with higher fatigue and yield strength. The ultimate tensile strain as well as the yield strength are normalised by the fatigue strength of position A at ten million load cycles.

### 3.2. Fractography

Furthermore, all tested fatigue specimens are analysed by means of fractographic analysis. In order to do so, the crack-initiating inhomogeneity of every tested specimen is determined and subsequently geometrically characterised. Amongst others, the effective projected area of the most critical defect perpendicular to the load direction as well as the minimal and maximal distance to the surface are investigated by means of scanning-electron and digital optical microscopy. Further information about this methodology is given in [34]. Subsequently, the relevant geometrical characteristics, such as the equivalent circle diameter $d_{eq}$, are derived (see Equation (3)).

**Figure 8:**
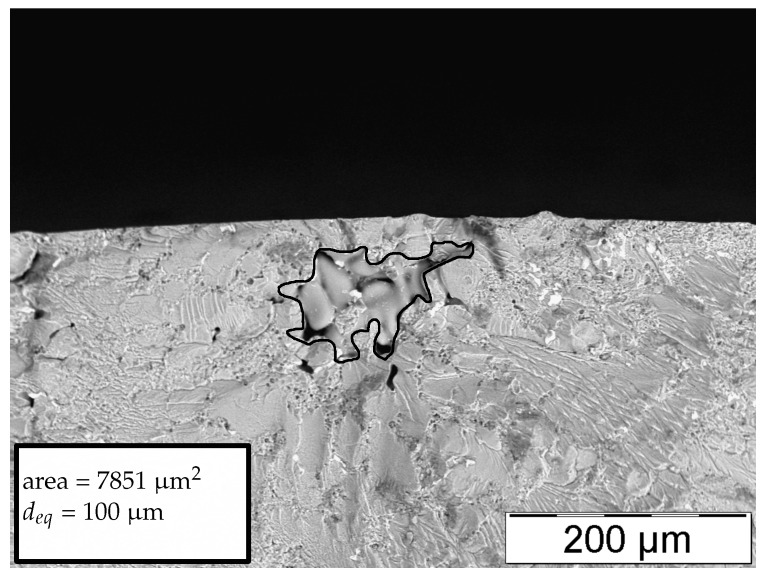
Representative fatigue fracture-initiating pore of position A.

**Figure 9:**
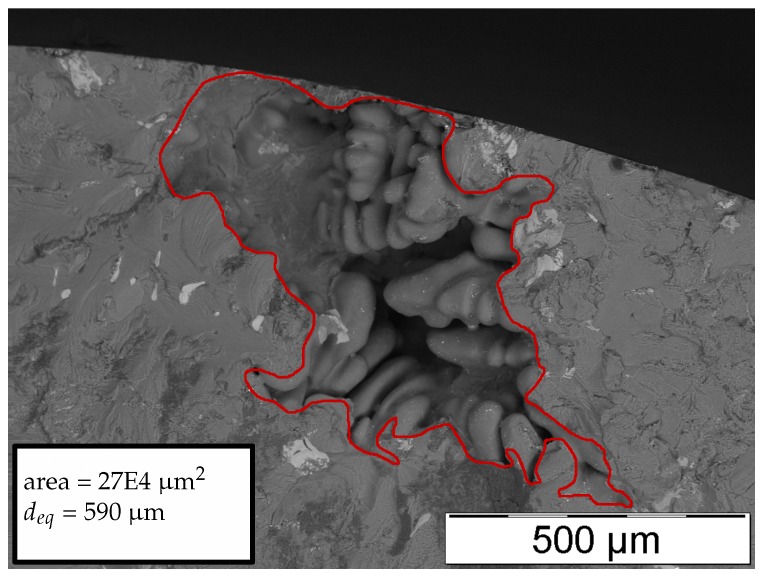
Representative fatigue fracture-initiating pore of position B.

**Figure 10:**
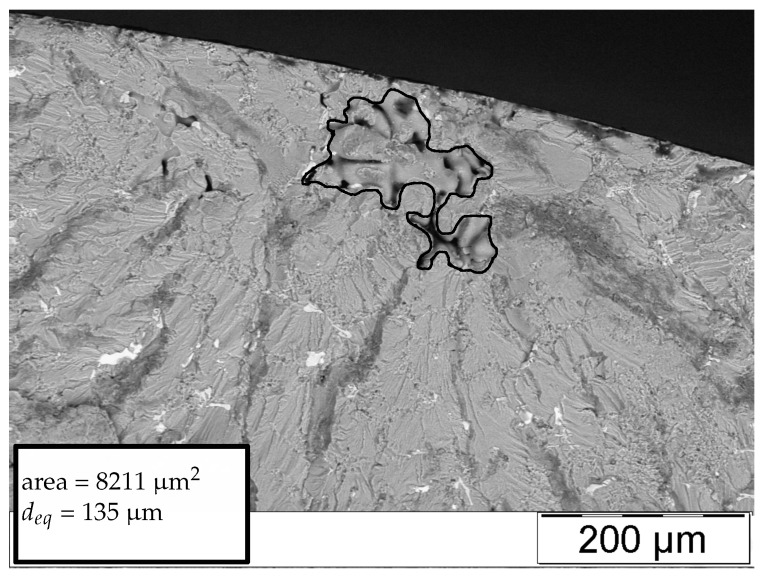
Representative fatigue fracture-initiating pore of position A.

**Figure 11:**
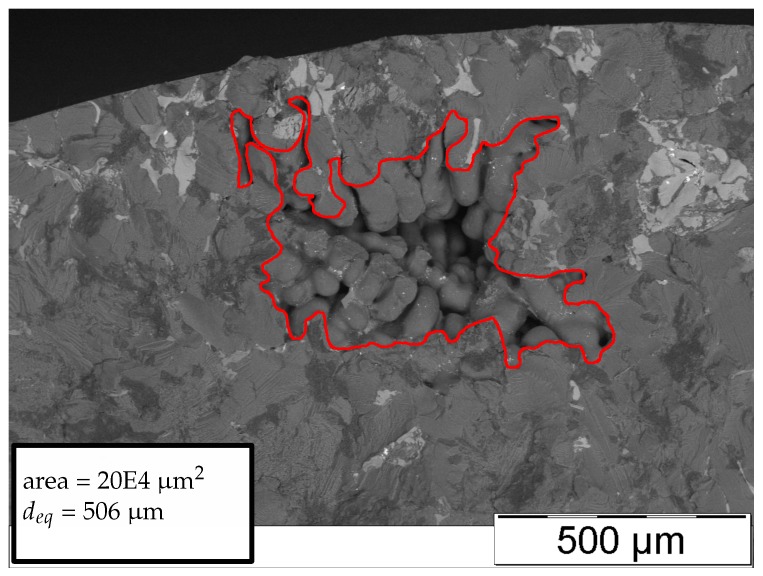
Representative fatigue fracture-initiating pore of position B.

**Figure 12:**
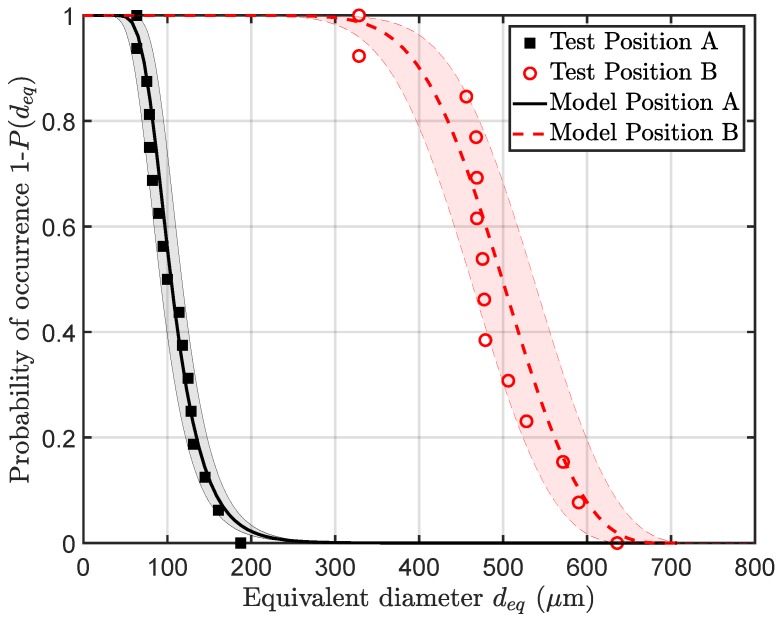
Probability of occurrence of defects depending on equivalent circle diameter $d_{eq}$.

Figure 8, Figure 9, Figure 10 and Figure 11 display representative fracture-initiating defects at sampling positions A and B. Following up, the statistical distribution of the very extremal defects are deduced by means of a maximum-likelihood function fit [62]. As suggested by preliminary studies in [12,14], the cumulative distribution of the extreme value distribution type 1 is utilised, to describe the extremal flaws (see Equation (13)). This methodology is invoked, as it is proposed to estimate the parameters of the distribution best by [63]. In conclusion, the probabilities of occurrence of critical inhomogeneities is derived (see Figure 12). Figure 12 depicts not only the probability of occurrence of the experimental data but also the model based on the evaluated distribution of each sample position. In addition, the 90% confidence band of the location parameter is displayed.

Therefore, a defect with an equivalent circle diameter $d_{eq}$ of around 100 μm may occur with a probability of 50% in position A, whereas, for position B, a significantly greater defect with a size of about 500 μm may occur for the same probability.

### 3.3. Fracture Mechanical Tests

In order to set up a defect based fatigue assessment model, it is of utmost importance to evaluate the crack propagation behaviour of the investigated material. Therefore, fracture mechanical tests are conducted. The specimens are slit prior to the actual crack propagation test by means of a custom device. Next, all specimens were compression pre-cracked, in line with a recommended methodology given in [64,65,66,67]. Herein, the single edge notched bending (SENB) specimens were oscillated for a total number of 400,000 load cycles at a load ratio of *R* = 20 by means of a electromagnetic resonance testing machine. This procedure initiates an initial crack and builds up residual tensile stresses in the very notch root of the specimen. Afterwards, the SENB specimens are tested at an electromagnetic resonance bending test machine while in situ measuring of the direct-current driven potential drop. This methodology enables an in-line measurement of the actual crack length. The corresponding stress intensity factors were furthermore estimated by means of [68,69].

**Figure 13:**
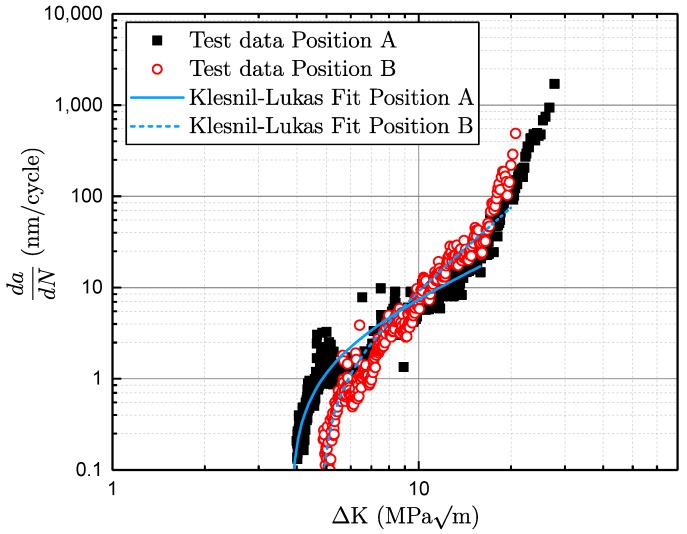
Representative crack-propagation curve with estimated Klesnil–Lukas [25] fit.

In Figure 13, a representative fracture mechanical test at a load ratio of *R* = −1 is displayed. Additionally, the coefficients of the Klesnil–Lukas approach [25] are estimated by means of a least-mean squares fit (see Equation (6)). In position A, three specimens are tested at a load ratio of *R* = −1, in order to cover a statistical variation of the test results. The fracture mechanical properties of specimen position B are taken from preliminary studies, which also include an enhanced number of specimens [70].

### 3.4. X-Ray Computed Tomography

In order to gain information about the defect population, it is essential to conduct X-ray micro-computed tomography (XCT) scans [20]. As the investigated sample possesses a small specimen diameter and a comparably low density, the transmission of X-rays enables a high resolution of at least 5.5 μm voxel-size. Hence, intrinsic flaws with a spatial extent of about 15 μm can be properly investigated. Experiences with computed tomography in material science are summarized in [71]. As the original approach from Tiryakioglu invokes the number of cycles for crack initiation $N_{i}$, the investigation by means of computed tomography enables an experimental in-depth evaluation of this value (see Equation (2)). Therefore, additional specimens are taken out from sampling position A and are incrementally tested in terms of high-cycle fatigue at a constant load amplitude. The normalised stress amplitude is defined to be 1.21, such that the specimen is loaded definitely within the finite-life region. After a given number of cycles, the fatigue test is stopped, and the specimen is investigated by means of a XCT scan. The scans are conducted at a predefined inspection interval of $N_{scan,i+1}=2\times N_{scan,i}$ until specimen failure occurs.

**Figure 14:**
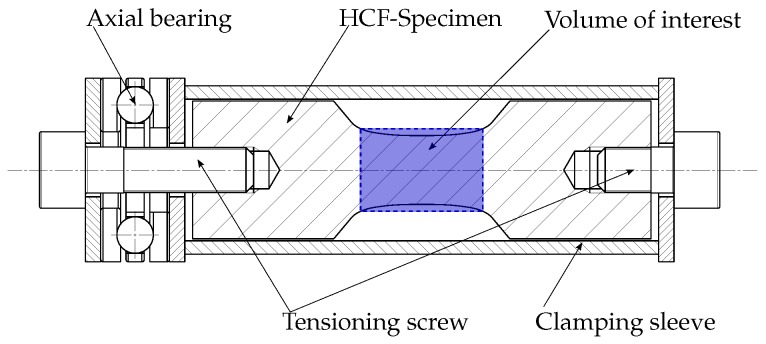
Clamping device for computed tomography analysis.

In order to open the cracks during XCT scanning, a special clamping device is developed. This clamping device enables the specimen to be investigated by computed tomography while being under static pre-tensioning, as recommended in [51]. Hence, the intrinsic cracks are unclenched, causing enough reflection to be recognized as an inhomogeneity by the detector. In addition, the aluminium clamping sleeve turns out to act as a pre-filter, such that it enhances the gained data quality in terms of less scan artefacts (see [72]). In order to apply a repeatable static stress in the specimens, a torque wrench is used. Furthermore, an axial bearing is installed in order to minimise torsion loading in the specimen itself. Figure 14 displays a schematic drawing of the utilised clamping device.

**Figure 15:**
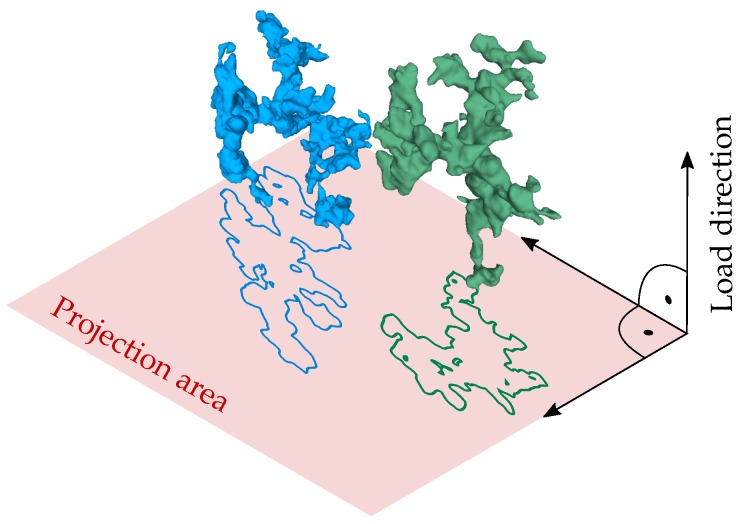
Representative XCT evaluated defects.

**Figure 16:**
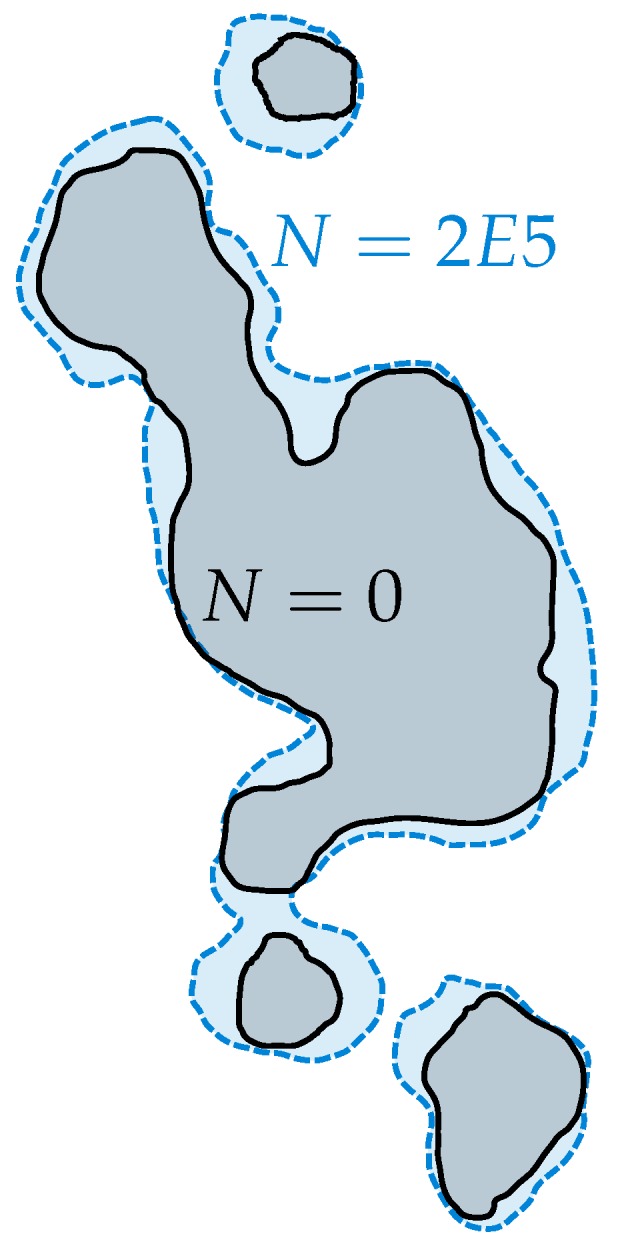
Evaluated crack propagation based on in situ XCT after 2E5 load cycles.

Figure 15 depicts two representative defects evaluated from the XCT scans with a spatial extent of approximately 64 μm. In order to evaluate the number of load cycles until crack initiation, the projected area perpendicular to the load direction is utilised. In addition, Figure 16 displays the projected area of a representative defect before testing at *N* = 0 and at *N* = 2E5 load cycles. The investigation reveals a rather rapid merging of detached flaws, forming a cluster of conjoined inhomogeneities, which leads to significantly enhanced projected pore sizes.

**Figure 17:**
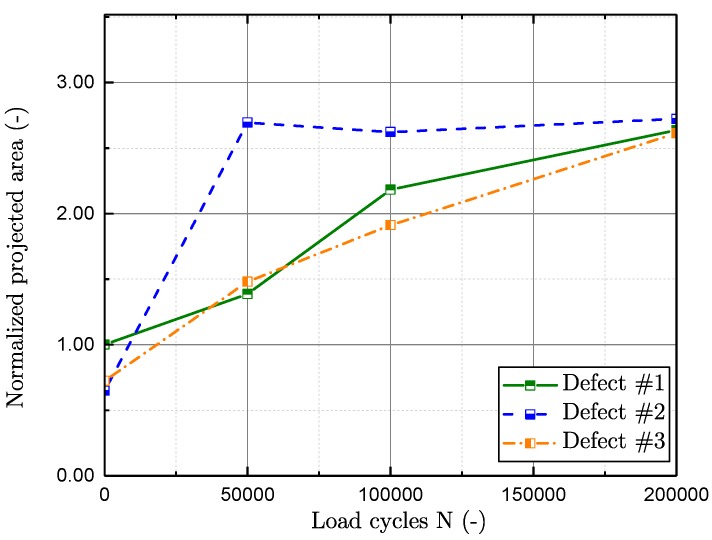
Projected area of representative defects over load-cycles.

**Figure 18:**
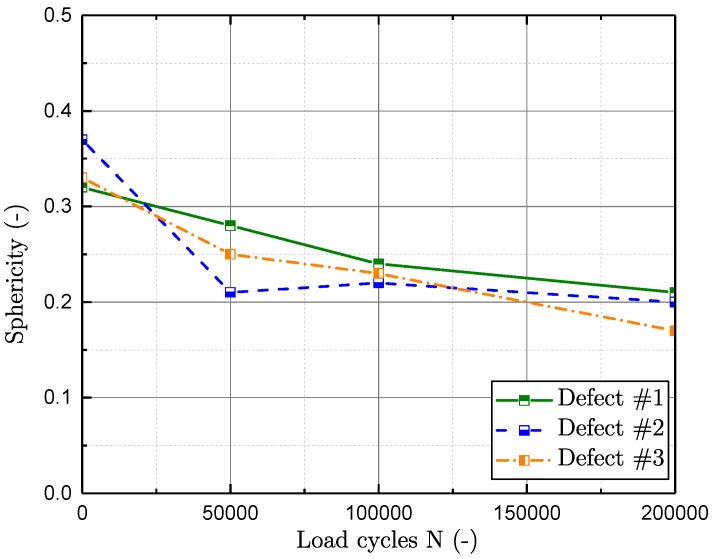
Sphericity ψ of representative defects over load-cycles.

In Figure 17, the evaluated spatial extent, by means of the normalised projected area perpendicular to the load direction, of representative flaws is displayed over the number of load cycles. The projected area of the three flaws is normalised by the projected area of the greatest defect at zero load cycles. In Figure 17, the three largest detected defects at a cycle number of *N* = 0 are displayed. Defect #2 proposes a significant crack propagation immediately after the very first inspection interval. Nevertheless, the crack turns out to be non-propagating according to the subsequent investigations. On the other hand, the evaluation of defects #1 and #3 reveal a proper crack propagation rate with increasing load cycles. The investigated results propose the number of cycles until a crack initiates to propagate $N_{i}$ thus to be between zero and about 10% of the whole fatigue life, which is in line with preliminary studies [35,73]. Figure 18 displays the decrease of sphericity ψ of intrinsic flaws with enhanced load cycles. The sphericity rates the degree of circularity ranging from zero to one, where a value of one equals a sphere. The sphericity ψ is evaluated by means of Equation (9), and hence defines a ratio of the surface $A_{pore}$ of a defect with volume V with a spherical surface with equal volume. The decrease agrees with the assumption that a sharp crack initiates at an inhomogeneity after $N_{i}$ cycles.

(9) $$\psi =\frac{\pi^{\frac{1}{3}}{\left(6\cdot V_{pore}\right)}^{\frac{2}{3}}}{A_{pore}}.$$

Hence, after initiation of a crack at the most-stressed inhomogeneity, the free surface of the flaw $A_{pore}$ is greatly enhanced, whereas the very pore volume $V_{pore}$ is increased by only a small amount. For comparison, representative flaws of aluminium castings with varying sphericity ψ can be found in [39].

### 3.5. Fatigue Assessment Model

In order to estimate the high-cycle fatigue behaviour, it is necessary to characterise the crack propagation both in regions 1 and 2. Therefore, the formula of Lukas and Klesnil [25] is utilised, see Equation (6), whereas the stress intensity range ΔK can be written as

$$\Delta K=2Y\sigma_{a}\sqrt{\pi a}.$$

After reinsertion in Equation (6) and a subsequent integration this leads to Equation (10):

(10) $$N_{f}=N_{i}+A_{i}^{\frac{2-m}{4}}\cdot B\cdot \sigma_{a}-\tilde{C}\cdot \Delta K_{th,lc}^{-m},$$

with

(11) $$\tilde{C}=\frac{1}{m-2}\left(\frac{2}{C}\right).$$

Hence, the number of load cycles until fracture $N_{f}$ can be assessed utilising Equation (10). According to preliminary studies [21], the number of load cycles for initiation of an incipient crack $N_{i}$ is correlated to the total number of cycles until fracture $N_{f}$. The long crack threshold $\Delta K_{th,lc}$, as well as the coefficients *m* and *C* are evaluated by a least-mean square fit of the fracture mechanical test data, invoking Equation (6). In addition, due to the formula being valid also in the region 1 of the crack propagation curve, the endurable stress amplitude can be easily evaluated for a given number of cycles (see Equation (12)):

(12) $$\sigma_{a}={\left(\frac{N_{f}-N_{i}+\tilde{C}\Delta K_{th,lc}^{-m}}{A_{i}^{\frac{2}{2-m}}B}\right)}^{-\frac{1}{m}}.$$

In order to validate this lifetime model, the fatigue data from the tested specimens is utilised. Furthermore, the long crack threshold as well as the evaluated parameters from the Klesnil–Lukas fit for regions 1 and 2 of the crack propagation curve are taken both from fracture mechanical tests and preliminary studies [70]. First, all tested specimens are projected by means of a bilinear model from the finite-life region to the long-life region. Therefore, all displayed fatigue data refer to the projected stress amplitude at a total number of ten million cycles.

**Figure 19:**
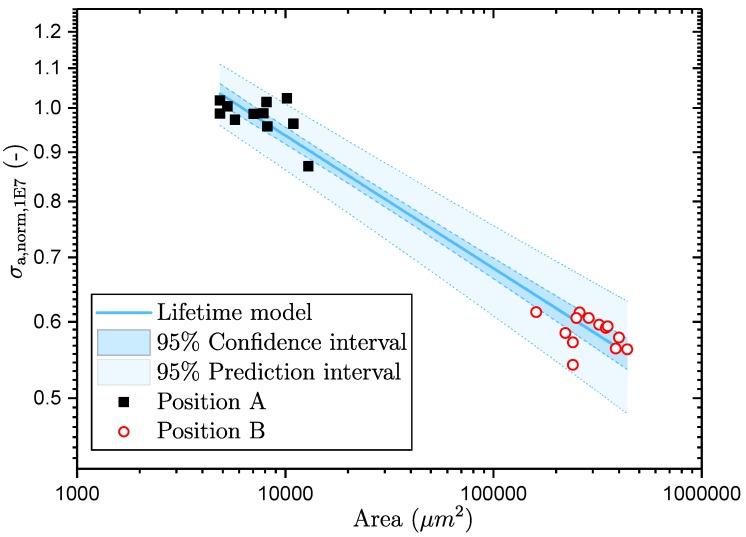
Defect based, probabilistic fatigue assessment model.

Afterwards, the Levenberg–Marquardt iteration algorithm is utilised to minimise the least-squares errors [74]. Thus, the parameters *m* and *B* of the lifetime model can be obtained by iterative adjustment of the initial variables. Figure 19 displays both the modified fatigue lifetime model from Equation (12) as well as the projected testing data from the investigated sampling positions. In addition, the fit is statistically evaluated by means of computing the coefficient of determination $R^{2}=0.97$, which suggests that the model matches well to the test data. Additionally, the 95% confidence and prediction intervals for the fit parameter *B* are plotted in order to visualise the acceptable correlation.

In order to characterise the statistical distribution of the spatial extent of fracture initiating defects, the type-one extreme value distribution is applied. The cumulative density function of the Gumbel distribution is gained by integration of Equation (1) (see Equation (13)). By substitution of the defect area $A_{i}$ with the statistical distribution of the equivalent circle diameter $d_{eq}$, the influence of extremal pore size distribution on the fatigue lifetime can be assessed:

(13) $$P=\exp\left[-\exp\left(\frac{d_{eq}-\mu }{\delta }\right)\right],$$

which leads after restructuring to

(14) $$d_{eq}=\mu +\delta \left\{-ln\left[-ln(P)\right]\right\}.$$

The initial area of the fracture initiating defect $A_{i}$, can be equated by the probability of occurrence of an equivalent circle diameter $d_{eq}$ squared and multiplied by $\frac{\pi }{4}$. Thus, the probability of survival, depending on the statistical distribution of defect sizes, for both finite-life and long-life fatigue regions can be expressed as Equation (15):

(15) $$P_{s}=\exp\left\{-\exp\left[\frac{\mu }{\delta }-\frac{2}{\delta \sqrt{\pi }}{\left(\frac{N_{f}-N_{i}+\tilde{C}\Delta K_{th,lc}^{-m}}{B\cdot \sigma_{a}^{-m}}\right)}^{\frac{2}{2-m}}\right]\right\}.$$

**Figure 20:**
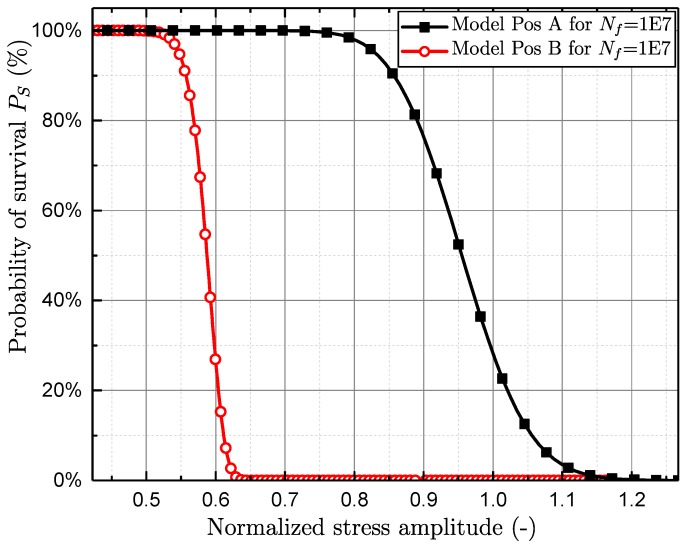
Probabilities of survival for investigated specimen positions A and B.

Figure 20 displays the estimated probabilities of survival for both investigated specimen positions A and B. In order to estimate the probabilities of survival $P_{s}$, the number of cycles until failure $N_{f}$ was set to the runout number of ten million cycles. In addition, the number of load cycles for crack initiation $N_{i}$ was conservatively set to zero for both sampling positions due to the evaluated crack initiation results from computed tomography investigations. Therefore, the local DAS and defect size distribution greatly affects the endurable stress amplitude.

In order to validate the fatigue assessment model, both the finite-life and long-life regions are investigated. In the long-life region, the mean deviation between the fatigue strength of the lifetime model and the evaluated fatigue strength from the experimental data is in the range of 4% (see Table 4). In the finite-life region, two different load levels of each sample position were investigated. At position A, the normalised load levels are 1.47 and 1.26. Due to the significantly lower fatigue strength of position B, the normalised finite-life load levels are defined as 0.95 and 0.74.

**Figure 21:**
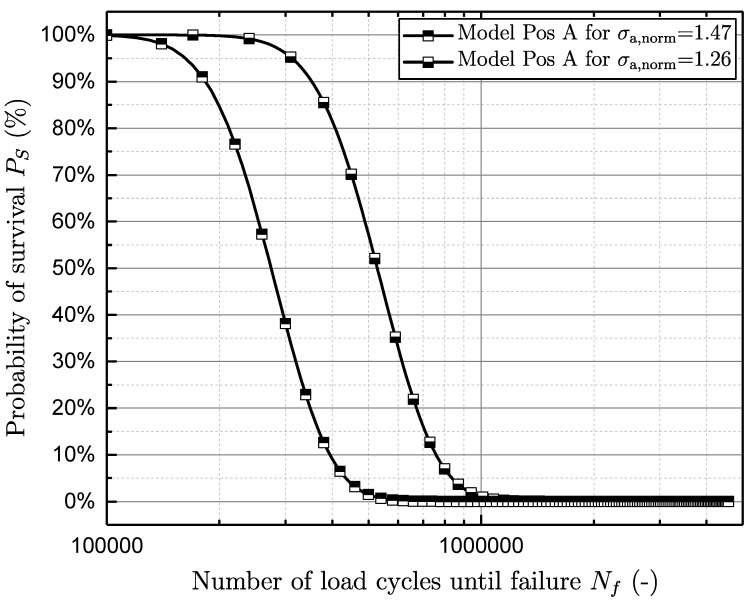
Validation of the model in the finite-life region position A.

**Figure 22:**
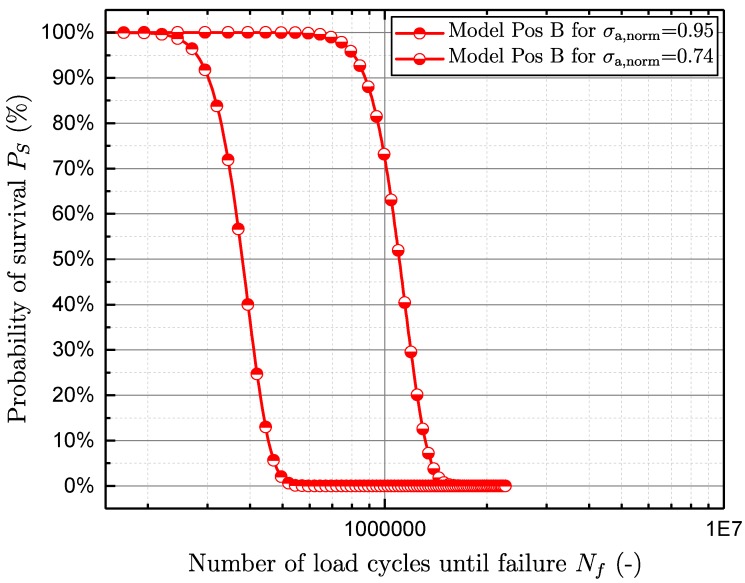
Validation of the model in the finite-life region position B.

Afterwards, the probability of survival is computed by Equation (15), by applying the very load level as stress amplitude and the number of load cycles until failure as a control variable. Figure 21 and Figure 22 show the validation of the model in the finite-life region of specimen positions A and B. In addition, the scatter band in the finite-life region $T_{N}$ both from experimental test data and from the fatigue assessment model at different load levels is calculated (see Table 5). It is shown that the scatter band in the long-life region $T_{S}$ of the model matches the experimental data well, whereas the model scatter band in the finite-life region $T_{N}$ underestimates the experimental data by an average of 10%.

## 4. Discussion

The presented fatigue approach is valid in the first and second crack propagation regime due to the implemented crack propagation model of Klesnil–Lukas. Therefore, not only the endurable stress amplitude in the finite-lifetime region, but also within the long-life region can be estimated. A defect correlated failure is mandatory in order to evaluate the spatial extent of the initiation crack $A_{i}$. By implementation of the statistical pore size distribution, the probabilities of failure and survival can be utilised. Furthermore, in situ computed tomography investigations proposed that the number of cycles for crack initiation is almost negligible for the investigated material.

In line with existing defect-based fatigue strength approaches, the upper lifetime boundary of the model has to be evaluated, since the fatigue strength of even defect-free material will be limited by other influencing factors, such as intrinsic heterogeneities. Thus, future work will focus on the assessment of near-defect-free material, which will be accomplished by applying a hot isostatic pressed (HIP) post treatment.

Another research topic is the correlation between the statistical distribution of XCT evaluated pore sizes and the distribution of fatigue fracture initiating ones. Ongoing work proposes functions to weight the non-destructively evaluated defect population with numerically estimated geometry factors, such that the most critical flaws will not be assessed only by their size but also by their spatial orientation, shape and location in terms of minimal surface distances. Another interesting approach is proposed by the authors in [48] which takes the mean value of the evaluated 10% fraction of extremal pore sizes from XCT-scanning into account, which will be compared to subsequent studies.

As soon as the non-destructively investigated defect population properly meets the distribution of fatigue fracture initiating flaw sizes, the presented approach in this study can be invoked to get the defect correlated fatigue strength behaviour. Finally, the in situ computed tomography investigations will be extended in order to evaluate the crack initiation at local material inhomogeneities in detail.

## 5. Conclusions

Based on the results presented within this paper, the following conclusions can be drawn:

- Two sampling positions with varying local cooling conditions are investigated. Therefore, the local dendrite arm spacing as well as the defect population differ significantly and broadens the application field for the developed methodology. These changes reflect in the investigated quasi-static properties, revealing that position B possesses a relatively brittle material behaviour with a fracture elongation of just 0.15% compared to other sample positions.
- Extensive fatigue tests are conducted in the high-cycle regime. The fatigue data is statistically evaluated. Hence, the normalised fatigue strength of position A is set as unified reference value with a mean defect size of 100 μm. Furthermore, the evaluated normalised fatigue strength of position B is about 0.58 with an average defect size of 500 μm.
- X-ray computed tomography scans during intermittent fatigue testing revealed that the crack initiation lifetime is below 10% of the number of load cycles until burst failure for Al-Si-Cu cast alloys. This is in agreement with preliminary findings [35].
- Tiryakioglu’s fatigue lifetime model by [12] is modified in order to extend its area of validity towards the long-life region, utilising the crack propagation approach of Klesnil–Lukas [25]. The presented model highlights a proper correlation to the examined fatigue tests resulting in a deviation of the mean long-life fatigue strength of up to 5% and up to 9% in the finite-life region.

## Figures and Tables

**Table 1:** Nominal chemical composition of the investigated cast material.

Si [%]	Cu [%]	Fe [%]	Mn [%]	Mg [%]	Ti [%]
7.5–8.5	2.0–3.5	0.8	0.15–0.65	0.05–0.55	0.25

**Table 2:** Statistically evaluated fatigue data.

Position	$\sigma_{\mathrm{LLF},\mathrm{norm}}$ [-]	$k_{1}$ [-]	$k_{2}$ [-]	$N_{k}$ [-]	$1:T_{S,\mathrm{LLF}}$ [-]	$1:T_{N,\mathrm{FL}}$ [-]
A	1.00	5.06	25	1.1E6	1.21	2.46
B	0.58	4.55	23	2.7E6	1.13	1.5

**Table 3:** Statistically evaluated quasi-static data.

Position	${\mathrm{UTS}}_{\mathrm{norm}}$ [-]	${\mathrm{YS}}_{\mathrm{norm}}$ [-]	A [%]
A	3.52 ± 0.07	1.63 ± 0.04	1.62 ± 0.4
B	2.28 ± 0.10	1.38 ± 0.03	0.15 ± 0.04

**Table 4:** Validation of the fatigue assessment model in the long-life region (LLF).

	**Position**	$\sigma_{\mathrm{LLF},\mathrm{norm},\mathrm{Ps}50}$ **[-]**	$1:T_{S,\mathrm{LLF}}$ **[-]**
Experiment	A	1.00	1.21
Model	A	0.96	1.23
Deviation	A	−4.6%	+1.4%
	**Position**	$\sigma_{\mathrm{LLF},\mathrm{norm},\mathrm{Ps}50}$ **[-]**	$1:T_{S,\mathrm{LLF}}$ **[-]**
Experiment	B	0.58	1.13
Model	B	0.59	1.10
Deviation	B	+1.8%	−2.9%

**Table 5:** Validation of the fatigue assessment model in the finite-life region (FL).

	**Position**	$N_{f,\mathrm{Ps}50,\sigma_{a,\mathrm{norm}}=1.47}$ **[-]**	$N_{f,\mathrm{Ps}50,\sigma_{a,\mathrm{norm}}=1.26}$ **[-]**	$1:T_{N,\mathrm{FL}}$ **[-]**
Experiment	A	2.5E5	5.5E5	2.46
Model	A	2.8E5	5.3E5	2.14
Deviation	A	+9.1%	-3.8%	−14.9%
	**Position**	$N_{f,\mathrm{Ps}50,\sigma_{a,\mathrm{norm}}=0.95}$ **[-]**	$N_{f,\mathrm{Ps}50,\sigma_{a,\mathrm{norm}}=0.74}$ **[-]**	$1:T_{N,\mathrm{FL}}$ **[-]**
Experiment	B	3.8E5	1.2E6	1.57
Model	B	3.8E5	1.1E6	1.50
Deviation	B	0.0%	−8.6%	−4.7%

## Abbreviations

The following abbreviations are used in this manuscript:

GEV	Generalized extreme value distribution
μ,δ	Location and scale parameter of extreme value distribution type 1
$P_{f}$	Probability of failure
$N_{f}$	Number of cycles until failure
$N_{i}$	Number of cycles of crack initiation
B	Offset parameter
m	Slope parameter
$\sigma_{a}$	Stress amplitude
$\sigma_{n,a}$	Nominal stress amplitude
$\sigma_{LLF}$	Long-life fatigue strength amplitude
$d_{eq}$	Equivalent circle diameter
SEM	Scanning electron microscopy
XCT	X-ray computed tomography
$k_{\sigma }$	Stress concentration factor
$C_{0},k_{0},\alpha ,\beta$	Constants for number of cycles until crack initiation $N_{i}$
DAS	Dendrite arm spacing
C	Crack propagation parameter
ΔK	Stress intensity factor range
$\Delta K_{th,lc}$	Long crack threshold range
n	Coefficient for crack propagation
f	Crack opening function
R	Load stress ratio
p,q	Exponents of Forman–Mettu NASGRO equation
SENB	Single edge notched bending specimen
HCF	High-cycle fatigue
HV	Vickers hardness
$C_{1}$,$C_{2}$	Coefficients of *√*area approach
R	Load stress ratio
$k_{1}$	Slope of S–N curve in the finite-life region
$k_{2}$	Slope of S–N curve in the long-life region
$N_{f}$	Number of load-cycles until specimen burst failure
$N_{k}$	Number of load-cycles at transition knee point of S–N curve
$T_{S,LLF}$	Scatter band of S–N curve in the long-life regime
$T_{N,FL}$	Scatter band of S–N curve in the finite-life regime
${\mathrm{UTS}}_{\mathrm{norm}}$	Normalised ultimate tensile strength
${\mathrm{YS}}_{\mathrm{norm}}$	Normalised yield stress
A	Elongation at fracture
ψ	Sphericity
$V_{pore}$	Volume of a defect
$A_{pore}$	Surface of a defect
$\tilde{C}$	Crack propagation coefficient
$A_{i}$	Initial defect area
a	Crack length
$R^{2}$	Coefficient of determination
*P*	Cumulative density function of the Gumbel distribution
$P_{s}$	Probability of survival
HIP	Hot isostatic pressed
